# A retrospective study of cumulative absolute reduction in axial length after photobiomodulation therapy

**DOI:** 10.1186/s12886-024-03427-4

**Published:** 2024-04-25

**Authors:** Kaikai Qiu, Coveney David, Ying Li, Zhou Lei, Liyang Tong, Wen Lin

**Affiliations:** 1 Fuzhou Southeast Institute of Visual Ophthalmology, Fuzhou (City), China; 2 Firstwest Innovations, 350007 Perth (City), Australia; 3 The Affiliated Xuzhou Municipal Hospital of Xuzhou Medical University, Xuzhou (City, China; 4 Department of Optometry, Ningbo Eye Hospital of Wenzhou Medical University, 315000 Ningbo(City), China

**Keywords:** Axial length, Myopia, Photobiomodulation therapy, Retrospective, Children

## Abstract

**Background:**

To assess the age and timeline distribution of ocular axial length shortening among myopic children treated with photobiomodulation therapy in the real world situations.

**Methods:**

Retrospective study of photobiomodulation therapy in Chinese children aged 4 to 13 years old where axial length measurements were recorded and assessed to determine effectiveness at two age groups (4 ∼ 8 years old group and 9 ∼ 13 years old group). Data was collected from myopic children who received photobiomodulation therapy for 6 ∼ 12 months. Effectiveness of myopia control was defined as any follow-up axial length ≤ baseline axial length, confirming a reduction in axial length. Independent t-test was used to compare the effectiveness of the younger group and the older group with SPSS 22.0.

**Results:**

342 myopic children were included with mean age 8.64 ± 2.20 years and baseline mean axial length of 24.41 ± 1.17 mm. There were 85.40%, 46.30%, 71.20% and 58.30% children with axial length shortening recorded at follow-up for 1 month, 3 months, 6 months and 12 months, respectively. With respect to the axial length shortened eyes, the mean axial length difference (standard deviation) was − 0.039 (0.11) mm, -0.032 (0.11) mm, -0.037 (0.12) mm, -0.028 (0.57) mm at 1, 3, 6, and 12-month follow-up, respectively. Greater AL shortening was observed among the older group who had longer baseline axial lengths than the younger group (*P* < 0.001).

**Conclusions:**

Overall myopia control effectiveness using photobiomodulation therapy was shown to be age and time related, with the maximum absolute reduction in axial elongation being cumulative.

**Supplementary Information:**

The online version contains supplementary material available at 10.1186/s12886-024-03427-4.

## Background

Axial length elongation was commonplace in children, especially those myopia with fast progressing. Previously, the mean axial length was always reported elongation in the domain of myopia control of children [1–3]. Correspondingly, the efficacy to control the axial length elongation was often a percentage ratio, comparing the relative reduction in progression among different treatments [1–3]. Fortunately, photobiomodulation (PBM) therapy, was reported shortened axial length [4]. Based on perception of relative reduction at different follow-up timeline, we observed varying degrees of relative myopia control effectiveness over 100.00 − 121.05%, 130.43%, 113.89%, and 103.33% at intervals of 3 months, 6 months, 9 months, and 12 months, respectively using PBM therapy [4].

Axial length (AL) is a reliable metric for assessing myopic progression due to its objective nature and comparability between endpoint and baseline measurements. Interferometry-based AL measurements, as highlighted by Brennan et al., underscore axial elongation as the ideal endpoint in evaluating myopic progression.

To circumvent the issues associated with presenting therapy outcomes as relative effects and to address the challenge of reporting inconsistent therapy efficacy, we initiated this retrospective study. Our aim is to evaluate myopia control efficacy using Cumulative Absolute Reduction in Axial Elongation (CARE), as advocated by Brennan NA et al. [5], which we propose as the preferred effectiveness metric.

Cumulative Absolute Reduction in Axial Elongation (CARE) serves as a treatment outcome metric that quantifies the actual physical reduction in ocular axial length (AL) For this study, a successful myopia control rate is defined as having a value of CARE ≤ 0, where CARE = AL after therapy - baseline AL.

Since PBM therapy demonstrates capacity in achieving significant axial length (AL) shortening within a few months, notably in the first month for myopic eyes with longer AL, Cumulative Absolute Reduction in Axial Elongation (CARE) seems a rational outcome metric for assessing effectiveness in real-world situations. AL measurement is much quicker and more accurate than cycloplegic refraction for determining myopia control effectiveness, especially within the first month. Notably, CARE values less than 0 were common with PBM therapy in children with myopia.

However, real-world reports on PBM therapy for myopia control are scarce. It’s uncertain whether the effectiveness of PBM therapy in real-world applications matches that observed in randomised controlled trials (RCTs), which have strict inclusion and exclusion criteria. In the real world, patient variability, compliance levels, and differences in irradiance used in marketed devices contribute to the complexity of evaluating effectiveness. This variation prompted our study to determine whether real-world effectiveness, as measured by CARE, was more attributable to patient factors or product differences, such as varying irradiance levels. In practice, there’s a phenomenon where different device manufacturers use different irradiance levels without substantiating evidence to support which is optimal (for example 2.50mW vs. 0.35mW). Furthermore, some eye care professionals prescribing PBM therapy tend to recommend lower irradiance levels for younger patients with myopia, despite the lack of evidence supporting this approach.

## Methods

### Study design

This study was focusing on two distinct age groups undergoing PBM therapy for myopia control. It aimed to evaluate the differential effectiveness of PBM therapy across these cohorts at three centers: Tianjin Airdoc Clinics, Ningbo Eye Hospital, and the Affiliated Xuzhou Municipal Hospital of Xuzhou Medical University in China.

This retrospective study obtained the ethical approval by the Ethics Committee of Tianjin Airdoc Clinic (approval no. 2023–004) and was conducted in accordance with the 1964 Helsinki Declaration and its later amendments or comparable ethical standards. Informed consent requirements were waived by our Institutional Review Board because of the retrospective nature of our study.

Our data collection methods were as described by Wang W and et al. [6].

Data were collected for those subjects who underwent PBM therapy during 2020–2023 with documented follow-up and baseline records between January 1st, 2020 and July 1st, 2023.

Inclusion criteria were myopic children who underwent PBM therapy for at least 6 months, had clinical data available at both baseline and at least one follow-up visit at 1 month onwards, and aged between 4 and 13 years. Subjects must have been diagnosed with myopia, which was defined as spherical equivalent refraction (SER) of at least − 0.50 diopter (D), with complete AL data available.

Participants were excluded if they had binocular vision or other ocular abnormalities, systemic disease, or history of ocular surgery. Children who previously had myopia control treatments other than single-vision spectacles (such as atropine, orthokeratology, and multi-focus myopia control lenses), were also excluded.

### Photobiomodulation therapy

Devices for PBM therapy were purchased or provided free of charge for the duration of the study. The PBM therapy device was a portable, collapsible design and could be used as a consumer-controlled light therapy device, as supplied by Airdoc Seconee, Beijing Airdoc Technology Co., Ltd., Beijing, China. The device had class II medical device certification in China, had been widely used for amblyopia treatment, and has been used for myopia control since 2007.

In brief, the device contained Class I semiconductor laser diodes which emitted the low-level red light at a wavelength of 650 ± 10 nm according to GB7247.1-2012. Illumination was held within 200 lx ∼ 1500 lx incident at the corneal surface. Pupil size was generally about 2.0–2.5 mm during the 3-minute lighting session, whereby both eyes were exposed to the laser source simultaneously.

Participants and their parents/guardians were instructed to use the PBM therapy at home under strict supervision by the parents/guardians. Children used the device twice daily (two treatment sessions per day). There was an interval of ≥ 4 h between the two sessions.

### Ophthalmic examinations

AL measurement was conducted on each eye prior to cycloplegia using an IOL-Master (Carl Zeiss 500/700, Meditec, Oberkochen, Germany), Lenstar (Lenstar LS-900, Haag-Streit AG, Koeniz, Switzerland), Nidek AL-Scan (Nidek Co., LTD., Tokyo, Japan), or Suoer Ocular Biometry (Suoer Co., LTD., Tianjing, China) depending on the biometry devices that were available and used in each specific clinic. It was mandated that the same device used at baseline for each participant was to be used at follow-up.

Cycloplegia was induced using two drops of 0.5% tropicamide 5 min apart, three times (total 6 drops per eye) before autorefraction. Autorefraction was recorded for each eye using Topcon KR-8800 (Topcon, Tokyo, Japan), Nidek ARK-900 (Nidek Co., LTD., Tokyo, Japan), or Tomey Auto Refractometer RC-5000 (Tomey Corporation, Nagoya, Japan). Three readings in each eye were taken and averaged until the desired precision (0.25 D for spherical and cylinder power, 5° for axis) was achieved. Cycloplegic spherical equivalent refraction (SER) was defined as the spherical power plus half of the cylindrical power.

### Definition of myopia control effectiveness and AL shortening

The chosen effectiveness metric for assessing myopia control with PBM therapy was the change in axial length (AL), which relates to changes in AL was defined as Cumulative Absolute Reduction in axial Elongation (CARE). In PBM therapy, an effective CARE signifies AL at follow-up being shorter than AL at baseline, which means there was a cumulative absolute reduction in axial length over time.

A greater CARE value (CARE 4 for example) was preferable to a lower CARE value (CARE 1 for example). Myopia control effectiveness in terms of AL shortening was defined as an AL reduction of ≥ 0.00 mm/year (CARE 1), ≥ 0.05 mm/year (CARE 2), ≥ 0.10 mm/year (CARE 3) and ≥ 0.20 mm/year (CARE 4).

CARE 1 was the primary target outcome across all age groups, as this would demonstrate AL stability. A secondary target outcome was the magnitude of change in AL per year among those showing AL shortening based on CARE 2, CARE 3 and CARE 4.

Baseline age was categorized into two groups as follows: 4–8, and 9–13 years old. Myopia was classified as SER ≤-0.50D. We undertook this retrospective study primarily to evaluate the effectiveness of PBM therapy concerning axial length (AL). However, it’s worth noting that an essential inclusion criterion was a spherical equivalent refraction (SER) of ≤ -0.50D at baseline. Irradiance was defined as two separate groups of 0.30mW and 1.20mW.

### Statistical analysis

Changes in AL at Month 1, Month 3, Month 6, Month 9 and Month 12 were calculated for the two age groups against irradiance, gender, and baseline age. Analysis was performed based on data both from the participant’s right eyes and the left eyes. Continuous and categorical variables were presented as mean [standard deviation (SD)] or number [percentage (%)], respectively. Frequency of AL shortening was presented under three criteria stratified by age, gender, and irradiance. The baseline parameters at both groups were compared with Chi-square or Kruskal–Wallis rank sum tests when appropriate. A polyline graph described the distribution, the maximum value of CARE, and the mean value of AL rate of change (CARE) at different follow-up. Multivariable logistic linear regression models investigated the relationships between AL shortening and other variable factors. The data was analyzed with the SPSS 22.0 software package, the hypothesis test was a two-tailed test with the α level set to *P* < 0.05. The measurement data in accordance with normal distribution and homogeneity of variance were tested via independent sample T test or ANOVA. Odds Ratio (OR) was calculated between the two groups considering CARE 1 values between the 0.37mW group and 1.20mW group.

## Results

Baseline Characteristics of Included Subjects as showed in Table 1.

**Table 1:** Baseline characteristics (*n* = 342)

Characteristics	Baseline	Minimum	Maximum
Gender (male: female)	193:149		
Age(yeas)	9.48 ± 10.93	4.36	12.88
SER(D)	-2.51 ± 0.99	-7.25	0.75
AL (mm)	24.44 ± 1.08	21.49	26.88
CARE (mm) at 12-month	-0.07 ± 0.07	-0.64	0.38
CARE (mm) at 1-month	-0.03 ± 0.18	-0.24	0.10

SER: spherical equivalent refractive error; AL: axial length; SD: standard deviation

### Frequency of CARE after PBM therapy

Due to the PBM therapy, 58.30%, 47.50%, 33.30%, and 10.00% children recorded AL shortening based on criteria of 0.00 mm/year (CARE 1), 0.05 mm/year (CARE 2), 0.10 mm/year (CARE 3), and 0.20 mm/year (CARE 4) respectively.

At Month 1, the CARE frequencies were 93.30% (CARE 1), 62.20% (CARE 2), 26.70% (CARE 3), and 2.20% (CARE 4).

The maximum values of AL shortening were seen at month 6 or month 12, while the maximum AL elongation was only seen at month 12. Furthermore, those individuals demonstrating AL shortening at month 1 due to PBM therapy showed greater CARE between month 6 and month 12. On the contrary, those with AL elongation at month 1 showed even longer AL at month 12.

Table 2 contains the frequency of CARE per year stratified by baseline age and gender. There was a significant increase in the frequency of AL shortening with increasing baseline age, baseline AL, at month 1 on AL shortening ≤-0.05 mm, -0.10 mm, -0.20 mm and 0.00 mm (all *P* < 0.05).

**Table 2:** Frequency of axial shortening (CARE values) at follow-ups of 12-, 1-, and 6- months

	*N*	CARE1	CARE2	CARE3	CARE4
**Total at Month 12**	66	41(62.10%)	32(48.50%)	21(31.80%)	8(12.10%)
**Total at Month 1**	45	42(93.3%)	28(62.20%)	12(26.70%)	1(2.20%)
P = value*		**< 0.001** ^*^	**< 0.001** ^*^	**< 0.001** ^*^	**< 0.001** ^*^
**AGE**	**N**	**CARE1**	**CARE2**	**CARE3**	**CARE4**
8	70	10 (22.7%)	8 (18.2%)	4 (7.6%)	0
9	58	13 (10.7%)	8 (6.6%)	3 (2.3%)	0
10	44	22 (18.6%)	10 (8.5%)	2 (1.6%)	0
11	22	37 (41.6%)	27 (30.3%)	3 (3.4%)	1(1.41%)
12	20	12 (60.0%)	10 (50.0%)	10(50.0%)	6(10.0%)
13	35	21 (60.0%)	14 (40.0%)	4 (11.4%)	3(8.6%)
P-value*		**< 0.001** ^*^	**< 0.001** ^*^	**0.025** ^*^	**0.385**
**Gender**	**N**	**CARE1**	**CARE2**	**CARE3**	**CARE4**
Girls	193	60 (30.0%)	41 (20.5%)	14 (7.0%)	7(3.6%)
Boys	149	55 (23.5%)	35 (15.0%)	6 (2.6%)	9(6.0%)
*P*-value*		0.098	0.447	0.343	0.377

*Bold indicates statistically significant

### Mean change of AL after PBM therapy

For CARE 1 (≤ 0.00 mm/year) the mean (SD) was − 0.03 (0.17) mm/year for all. The older age group was correlated with greater AL shortening than the younger age group (-0.07 ± 0.13 mm versus 0.06 ± 0.17 mm; *P* < 0.001), with the magnitude of AL shortening being − 0.137 (0.056) mm/year among children aged 9–13 years and + 0.271 (0.261) mm/year among children aged 4–8 years (*P* < 0.001) at Month 12. At Month 6, Month 3 and Month 1, both groups had all mean AL shortening, respectively (-0.02 ± 0.11 mm versus − 0.12 ± 0.13 mm, *P* < 0.001; -0.05 ± 0.09 mm versus − 0.12 ± 0.10 mm, *P* = 0.001; -0.03 ± 0.16 mm versus − 0.11 ± 0.06 mm, *P* = 0.017).

**Fig. 1:**
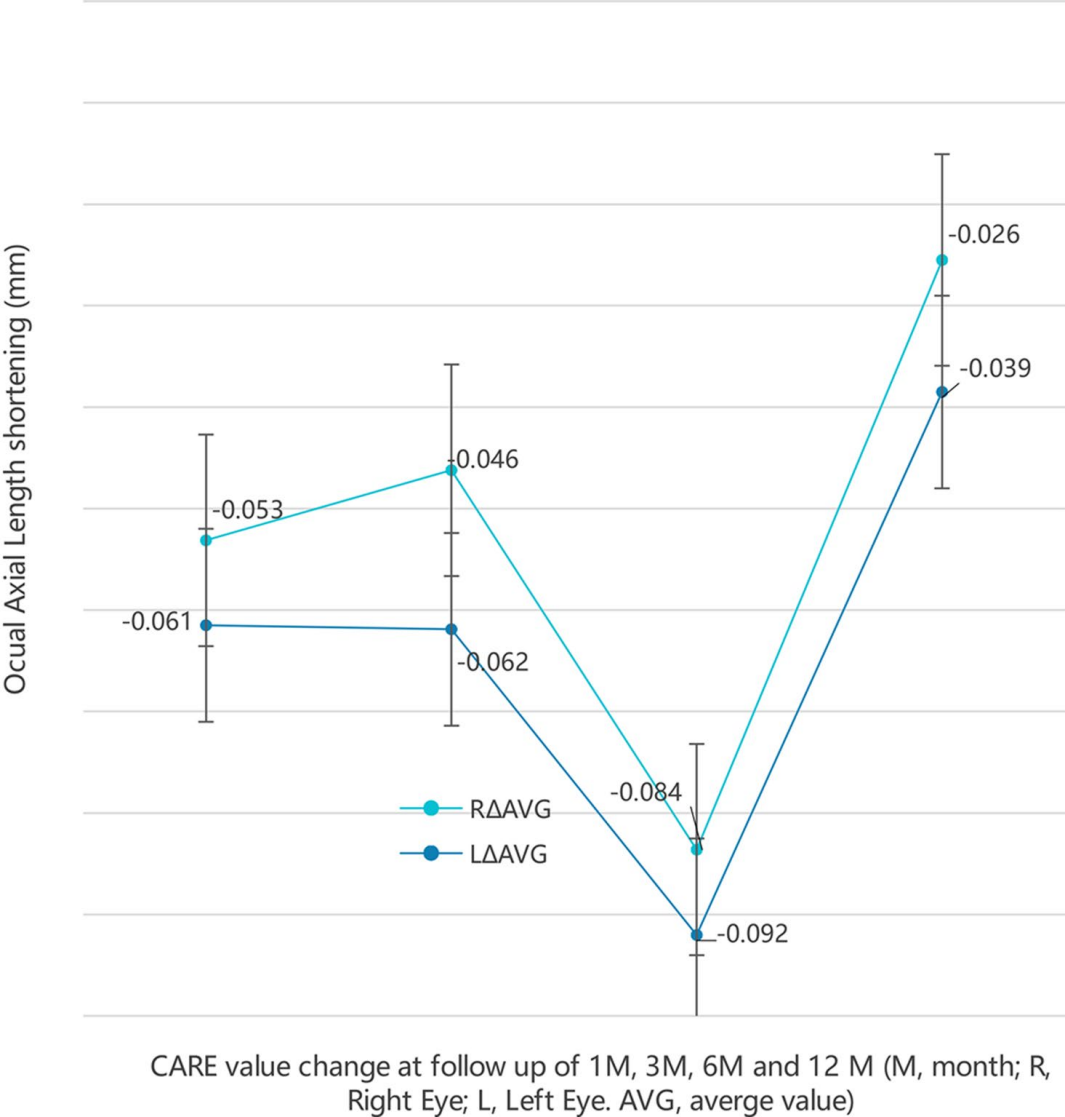
Change of CARE at follow-up

Figure 1 elucidated the relationship between mean AL changes of both the right and left eyes after PBM therapy with different duration. A significant increase in the CARE 4 was noted with increasing therapy duration. The maximum numbers of CARE 4 were achieved at Month 6 or Month 12 while the first month had the lowest percentage of CARE 4 at each age level. The magnitudes of AL shortening per year among those experiencing AL shortening were similar across gender (*P* = 0.707).

The AL parameters were closely correlated to spherical equivalence, ages and the CARE (*P* < 0.001, *P* = 0.013, *P* = 0.011), respectively.

**Fig. 2:**
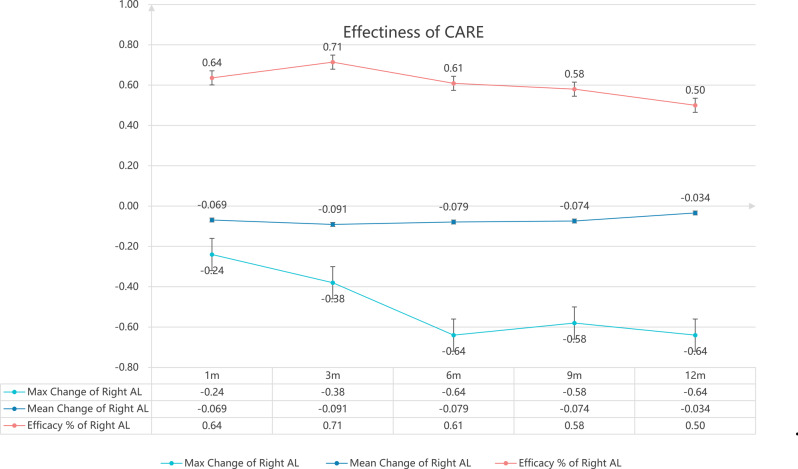
CARE and percentage of AL shortening after PBM therapy. Note: AL: Axial Length (mm); Max: maximum; %: Percentage

Figure 2 elucidated the distributions of AL shortening effectiveness, maximum and mean changing values of AL during the different follow-up timeline among all. And the maximum value of AL shortening reached the peak during the later follow-up timeline (such as Month 6 and Month 12) while the negative CARE values was more at the early follow-up timelines (such as Month 1 and Month 3).

## Discussion

With this retrospective data, myopia control effectiveness with PBM therapy varied at different follow-up intervals, with 59.10% of children attaining no AL elongation (CARE ≤ 0.00 mm per year) over 12 months. In addition, 8.70% of participants had AL shortening > 0.20 mm at 12 months. Those who underwent AL shortening due to PBM therapy had a mean change of − 0.02 mm per year. PBM effectiveness in this real-world study was similar to both our previous published randomized controlled trial [4], another retrospective study [6], post hoc analysis of a randomized trial [7], and better than other non-PBM therapy interventions (1–2, 8, 9–13).

In this real-world study, several novel findings emerged. Firstly, the cumulative effectiveness of PBM therapy for myopia control, as illustrated in Fig. 2, was noteworthy. Additionally, the maximum observed negative CARE value (-0.64 mm) indicated substantial axial length shortening. Moreover, the study identified age-related differences in the effectiveness of PBM therapy.

Comparisons with previous randomized controlled trials (RCTs) of PBM therapy revealed both similarities and differences. Consistent with prior RCTs (14, 15, 16, 17–18) significant AL shortening was observed within the first month. However, a notable departure was observed in the timing of maximum AL shortening. Unlike previous RCTs (4, 14, 15, 16, 17–18), where maximum AL shortening occurred within the first 1–3 months [4], in this real-world data, the maximum effect occurred at both Month 6 and Month 12, with Month 6 exhibiting particularly pronounced results.

The assessment of PBM therapy’s cumulative effect included considerations of both the percentage of AL shortening and the various CARE values. For instance, while AL shortening was common at Month 1 (58% of subjects), neither the values of CARE < -0.20 nor CARE < -0.10 were prevalent. These findings highlight the importance of evaluating both the magnitude and timing of AL shortening in understanding the effectiveness of PBM therapy for myopia control.

AL was measured by optical biometric devices, which defined AL as the distance from cornea to the retinal pigment epithelium. AL measurements might be affected by changes due to circadian fluctuations, however the success rate of myopia control judged by CARE values is unlikely to be influenced or explained by measurement error produced with optical biometry (general accuracy and repeatability was within 0.02 mm).

Older children tended to achieve more significant AL shortening from PBM therapy than younger children, presumed due to their slower AL growth rate and underlying baseline conditions of longer AL or greater myopic spherical equivalence.

From Fig. 2, the maximum CARE appeared at later follow-up while the maximum distribution occurred at later follow-up, which demonstrated that PBM therapy probably had a cumulative effect. Age-related characteristics illustrated that PBM therapy was widely effective in the early phases of treatment, however only a small percentage of subjects showed continued AL shortening over time, especially among those who obtained larger magnitude AL shortening at early follow-up.

Interestingly, it seemed that the later 12-months outcome could be predicted from the earlier 1–3 months follow-up CARE, as per reported by Wang W (6–7). Both the maximum AL shortening and the maximum AL elongation were cumulative through to the final follow-up.

Reports on AL shortening with CARE in clinical studies have been relatively scarce, except for studies focusing on PBM therapy or repeated low-intensity red light exposure using laser diode red light with a wavelength of 650 nm ± 10 nm, which has garnered attention within the global myopia control community over the past three years.

There remains loose consensus within various myopia control interests that AL cannot be shortened, AL cannot undergo continued shortening, or AL cannot be shorted beyond the range 0.02 mm to 0.20 mm. However, we have readily demonstrated some myopes could achieve outcomes of AL shortening at the values of -0.58 mm or -0.64 mm, which was concordant with another similar low-intensity red light therapy report which describes similar phenomena (6–7), although their lighting had 2–5 times greater irradiance but less frequency administration per week.

PBM therapy presents a strong relative value proposition, offering superior myopia control with more effective CARE outcomes at lower cost when compared to other modalities such as lenslet spectacles or contact lenses. Whilst orthokeratology had been reported to show very short periods of AL shortening within the earlier months of wearing [8], the AL shortening phenomena was less pronounced when compared to low-intensity laser therapy [14]. All other methods of myopia control, such as DIMS [9], Orthokeratoloy [10], ROMIO [11], Misight [12] and ATOM2^14^, resulted in AL elongation when comparing the relative AL change over time.

Although a few myopia studies report AL shortening, most AL shortening data were considered anomalies in the past due to their uncommon incidence and insignificance in analysis. It was surmised that AL shortening could be attributed to measurement errors or fluctuations. Our previous RCT [4] confirmed significant AL shortening which was too large in magnitude to be attributed to measurement errors or anomalies.

In this latest retrospective study, 84.40%, 81.70%, 73.00%, 65.50% and 59.10% of myopic children undergoing PBM therapy achieved AL shortening at the 1, 3, 6, 9, and 12-month follow-ups, respectively while the CARE 1 myopia control rate was 93.33%, 85.00%, 77.84%, 67.33% and 62.10% at the 1, 3, 6, 9, and 12-month follow-ups, respectively. The current study reinforces findings of the previous RCT [4] within a clinical setting and reports even higher frequency of AL shortening with 12 months treatment duration (> 0.05 mm in 48.5% of subjects).

It appears the inclusion of older age (≥ 9 years old) groups with longer AL and more myopia in the current study might somewhat explain the larger frequency of AL shortening found in comparison to that previously reported in our earlier RCT [4], where younger (with shorter AL and less myopia) and older (with longer AL and more myopia) ages were both excluded from previous inclusion criteria.

It has emerged that different ages result in different myopia control rates. Myopia progression varies with age and severity of myopia [19]. Younger age has been identified as a determinant for worse treatment efficacy.

The older age group demonstrated significantly greater myopia control effectiveness compared to the younger age group across all follow-up intervals, as indicated by CARE 1, CARE 2, CARE 3, and CARE 4. This finding aligns with the conclusions drawn by Wang W et al. (6–7) in their multivariable model, where they reported a significant association between AL shortening and baseline age, as well as longer baseline AL.

In general, younger age groups exhibit faster rates of AL growth compared to older age groups. Concurrently, older age groups present with significantly longer baseline AL and SER. These factors collectively contribute to the improved myopia control rates observed in PBM therapy studies among older age groups [15].

Indeed, older children exhibited significantly slower rates of axial length (AL) growth and longer baseline AL, factors that predispose them to experience greater myopia control through AL shortening via PBM therapy. Conversely, younger children may have been more prone to lower compliance with the PBM therapy regimen, potentially influencing the observed differences in myopia control effectiveness between age groups.

In other studies, such as the Defocus Incorporated Multiple Segments (DIMS) study [9] age was also a significant factor for myopia progression assessment and evaluation in both refractive progression and AL elongation.

Interestingly, we found the AL shortening was related to irradiance, although this only had statistical odds ratio significantly different between the 1.20mW irradiance group than to the 0.30mW irradiance group. Other study results found there was no significant different CARE or SER among 3 irradiance groups over 6 months’ duration [16].

In this study, the 0.37mW group had younger ages with shorter AL at baseline than the 1.20mW group, and a lower proportion of high myopic SER than that of mild myopic SER (2.00% versus 17.52%; *P* = 0.581). Further studies with larger sample sizes and behavioral data are needed to confirm whether irradiance has a dose-effect response.

In addition, with the median annual AL magnitude among AL-shortened myopes being − 0.03 ± 0.17 mm and − 0.01 ± 0.17 mm for the right and left eyes, respectively, it was difficult to hypothesize whether AL shortening might occur in a dose-dependent manner such that AL eventually returned to an emmetropic range with greater duration of use.

Another consideration was the possibility of AL instability among participants, considering they were not closely followed in this study. While it was possible that AL might fluctuate with PBM therapy, our previous trial which followed participants more closely indicated this did not occur [4]. Considering the many questions surrounding efficacy yet to be addressed, future trials with longer treatment duration should confirm whether PBM therapy had a dose-dependent response, and whether these changes were permanent throughout and after use.

It was unknown how PBM therapy shortens AL. Further experimental studies are needed to clarify the mechanisms of AL shortening from PBM therapy, which might lead to a more effective and targeted approach for AL modulation.

Our research highlighted PBM therapy as a promising therapy capable of shortening AL in approximately 50% of myopes within a follow-up time frame of months. With this level of myopia reversal or AL shortening, PBM therapy might possibly revert mild myopes to emmetropia; although the frequency and magnitude of AL shortening in mild myopes of younger age was not as dramatic as in older myopes.

This might be partly due to younger myopes having faster axial length growth rates, which had been demonstrated in the ROMIO study [11]. The ROMIO study concluded that axial elongation was correlated with the initial age of the subjects (*P* < 0.001); while our study found both axial elongation and axial shortening were correlated with initial age of the myopic children (*P* < 0.001).

Certainly, PBM has the potential to reduce vision-threatening complications of myopia considering its aptitude for treating myopes with longer AL at baseline, and the choice of treating younger myopes with longer AL might effectively prevent high myopia in children where it was once destined to develop. It should be noted that PBM therapy in studies outside of myopia control have found benefits with modulating duration, intervals wavelength, and energy fluence (20–21). Considering the infancy of PBM therapy for myopia control, the effect of such factors is yet to be explored but should be considered by future studies wishing to develop PBM therapy to its full potential.

The strengths of this study include its real-world clinically- derived data and larger sample size, inclusion of study participants with 12 months or greater follow-up data for analysis, and standardized measurements across each study site. The real-world data had similar efficacy and safety compared to our previous reported RCT [4].

Some limitations must also be acknowledged.

First, the children included in this study were all of Chinese ethnicity, which were known to have differing rates of myopia and ocular characteristics [22]. Further studies should seek to iterate these findings in multi-ethnic populations and differing geographic regions.

Second, this study design included much fewer younger myopes (4–8 years old) than those in the older 9–13 years old group with the younger age group having significantly shorter AL.

Third, there might have been better compliance in those children showing better myopia control results with more follow-ups. Additionally, the dropout at 12-months was much less than at 6- months, and the remaining children may have had better long-term compliance with the PBM regimen.

Finally, the per year AL reduction estimates were based on data gathered at 12-month’s follow-up, giving less than a 1-year sample size, and hence it is not appropriate to view this as an estimate of reduction that will take place in subsequent years.

For future development of PBM as a mainstay treatment option and understanding the long-term impacts of PBM on AL, a longer follow-up study with consideration towards the dose, duration, and rebound effect is required.

AL shortening was commonplace with PBM therapy, proving it an effective method of myopia control. It was demonstrated to reduce AL in myopic children at the first 1-month follow-up. AL shortening frequency varied during subsequent follow-ups. PBM therapy was shown to be a proven practical and effective treatment for managing AL changes with negative CARE. And importantly, the AL shortening effect benefit was accumulated beyond the first month.

We’d like to highlight recent studies using red light PBM in mainland China which have shown that using PBM for two 3-minute sessions daily can control myopic progression and prevent myopia onset, not only for children, but also for adults21, as well as those with high myopia (6–7). It has also been demonstrated that repeated red light therapy resulting in clinically significant ocular AL shortening of ≤ 0.10 mm or ≤ 0.05 mm at the end of month 1 was predictive of continuing effectiveness at the end of month 12, with high probability of retaining ocular AL shortening compared to those of baseline values, independent of baseline situations (6–7).

Moreover, even adult myopia reported similar efficacy [23]. And neither the choroidal thickness changes nor the cornea curvature could fully explain the negative CARE; that is, AL shortening and myopia recession after weeks of therapy [23]. Experiments either from Jiang Y et al. [17] reporting high illumination of 1600 lx or from Wang Y et al. [18] reporting an illumination range of about 1200–1800 lx suggested that the inhibitory effect on myopia may be partly due to higher ambient illuminance, not totally due to the wavelength or the color red only. But it might be more than that, our previous illumination was only 400 lx to cause the similar effect [4].

Based on studies in tree shrews, the dose-response effect was not obvious if the lighting was within 50 ∼ 600 lx [24]. Likewise, we recorded no significant difference of CARE between the group of 0.37mW and the group of 1.20mW at any follow-up timeline. Although smaller sample size and younger age were prescribed 0.37mW, and those younger age children had shorter AL at baseline, which could partly explain why the Odds Ratio was much lower than that with the 1.20mW group, the latter being prescribed to the older aged myopic children.

It might be preferential to prescribe 1.20mW to short AL younger myopes at baseline instead of low dose 0.37mW, although whether it might be better to consider a high dose at first dose over a lower initial dose in the younger myope was still a puzzle. The ideal starting dose and subsequent dosing for follow up or maintenance of AL effect is still under long term observation and more evidence is required to formulate clinically useful treatment protocols to ensure PBM therapy effectiveness. Perhaps AL shortening will continue in special cases whereby they may not encounter an endpoint, as we have previously observed and reported on an accumulated maximum continuing AL shortening of 1.03 mm within 10 months [4]. And we had detected some special eyes could have continued AL shortening more than 16 month.

For consistent dosing in PBM therapy, it seemed that maximum AL shortening rates could not be sustained beyond about 6 months, hence therapy duration between about 1 ∼ 6 month might be most effective. Whether we consider with or without accumulated effects of AL shortening for myopia control in general, or even if we consider treatment at some level for each individual, it is unknown how long the phenomena of AL shortening could persist and to which extent the AL will undergo continued shortening if myopic eyes continue PBM therapy for another year or 10 years. Today, it remains unclear whether repeated long term or short term direct illumination through pupils with low irradiance (0.35 ∼ 2.5mW)red light at wavelength of 650 nm ± 10 nm could have some harm or accumulated harm to the human retina or not. There had been a reported case with ellipsoid zone disruption resulting in reduced best correct visual acuity after 5 months’ repeated PBM therapy with 2.0mW ∼ 2.5mW irradiance according to the reported literature [25]. However, the ellipsoid zone recovered back to a healthy retinal structure and best correct visual acuity returned after stopping the therapy. We have little understanding as to whether the higher irradiance or the individual’s characteristics (such as highly suspected hidden Stargardt disease) were in some way causative, nor whether such reversal on cessation could be commonplace in possible future likewise cases.

Our focus was to learn about and understand more about different effectiveness at different follow-up time frames with PBM therapy in two age groups, and additionally to explore phenomena between those with eyeball shortening and those with eyeball elongation, hence we initiated this retrospective study.

## Conclusions

Control myopia progression with PBM therapy based on CARE was measurable. Maximum CARE was accumulated both AL shortening and elongation.

### Electronic supplementary material

Below is the link to the electronic supplementary material.

Supplementary Material 1

## Data Availability

Data is provided within the manuscript or supplementary information files.

## References

[1] Rappon J, Chung C, Young G, Hunt C, Neitz J, Neitz M, Chalberg T (2023). Control of myopia using diffusion optics spectacle lenses: 12-month results of a randomised controlled, efficacy and safety study (CYPRESS). Br J Ophthalmol.

[2] Bao J, Huang Y, Li X, Yang A, Zhou F, Wu J, Wang C, Li Y, Lim EW, Spiegel DP, Drobe B, Chen H (2022). Spectacle lenses with aspherical lenslets for myopia control vs single-vision spectacle lenses: a randomized clinical trial. JAMA Ophthalmol.

[3] Cooper J, Tkatchenko AV (2018). A review of current concepts of the etiology and treatment of myopia. Eye Contact Lens.

[4] Zhou L, Tong L, Li Y, Williams BT, Qiu K (2023). Photobiomodulation therapy retarded axial length growth in children with myopia: evidence from a 12-month randomized controlled trial evidence. Sci Rep.

[5] Brennan NA, Toubouti YM, Cheng X, Bullimore MA (2021). Efficacy in myopia control. Prog Retin Eye Res.

[6] Wang W, Jiang Y, Zhu Z, Zhang S, Xuan M, Chen Y, Xiong R, Bulloch G, Zeng J, Morgan IG, He M (2023). Clinically significant axial shortening in myopic children after repeated low-level red light therapy: a retrospective multicenter analysis. Ophthalmol Ther.

[7] Wang W, Jiang Y, Zhu Z, Zhang S, Xuan M, Tan X, Kong X, Zhong H, Bulloch G, Xiong R, Yuan Y, Chen Y (2023). Axial shortening in myopic children after repeated low-level red-light therapy: post hoc analysis of a randomized trial. Ophthalmol Ther.

[8] Swarbrick HA, Alharbi A, Watt K, Lum E, Kang P (2015). Myopia control during orthokeratology lens wear in children using a novel study design. Ophthalmology.

[9] Lam CSY, Tang WC, Tse DY, Lee RPK, Chun RKM, Hasegawa K, Qi H, Hatanaka T, To CH (2020). Defocus incorporated multiple segments (DIMS) spectacle lenses slow myopia progression: a 2-year randomised clinical trial. Br J Ophthalmol.

[10] Qi Y, Liu L, Li Y, Zhang F (2022). Factors associated with faster axial elongation after orthokeratology treatment. BMC Ophthalmol.

[11] CHO P (2012). Retardation of myopia in Orthokeratology (ROMIO) study: a 2-year randomized clinical trial. Invest Ophthalmol Vis Sci.

[12] Chamberlain P, Peixoto-de-Matos SC, Logan NS, Ngo C, Jones D, Young G (2019). A 3-year randomized clinical trial of MiSight lenses for myopia control. Optom Vis Sci.

[13] Chia A, Chua WH, Cheung YB, Wong WL, Lingham A, Fong A, Tan D (2012). Atropine for the treatment of childhood myopia: safety and efficacy of 0.5%, 0.1%, and 0.01% doses (atropine for the treatment of myopia 2). Ophthalmology.

[14] Xiong F, Mao T, Liao H, Hu X, Shang L, Yu L, Lin N, Huang L, Yi Y, Zhou R, Zhou X, Yi J. Orthokeratology and low-intensity laser therapy for slowing the progression of myopia in children. Biomed Res Int.;2021; 8915867. 10.1155/2021/8915867. 10.1155/2021/8915867 PMC7861936 33575355

[15] Lin ZH, Tao ZY, Kang ZF, Deng HW (2023). A study on the effectiveness of a 650-nm red-light feeding instrument in the control and slow the progression of myopia. Ophthalmic Res.

[16] Zhou W, Liao Y, Wang W, Sun Y, Li Q, Liu S, Tang J, Li L, Wang X. Efficacy of different powers of low-level red light in children myopia control. Ophthalmology. 2023;S0161–6420. 10.1016/j.ophtha. 10.1016/j.ophtha.2023.08.020 37634757

[17] Jiang Y, Zhu Z, Tan X, Kong X, Zhong H, Zhang J, Xiong R, Yuan Y, Zeng J, Morgan IG, He M (2022). Effect of repeated low-level red-light therapy for myopia control in children: a multicenter randomized controlled trial. Ophthalmology.

[18] Wang Y, Li X, Abudukeyimu K, Du W, Ning Y, Qi X, Hua N, Wei N, Ding G, Li J, Song L, Zhang Y, Qian X (2023). Low-power red laser treatment for anisometropic myopia control in children: a contralateral comparison study. Discov Med.

[19] Verkicharla PK, Kammari P, Anthony Vipin Das (2020). Myopia progression varies with age and severity of myopia. PLoS ONE.

[20] Geneva II (2016). Photobiomodulation for the treatment of retinal diseases: a review. Int J Ophthalmol.

[21] Ivandic BT, Ivandic T (2012). Low-level laser therapy improves visual acuity in adolescent and adult patients with amblyopia. Photomed Laser Surg.

[22] Zhang Y, Qiu K, Zhang Q (2020). Ametropia prevalence of primary school students in Chinese multi-ethnic regions. Strabismus.

[23] Liu G, Li B, Rong H, Du B, Wang B, Hu J, Zhang B, Wei R (2022). Axial length shortening and choroid thickening in myopic adults treated with repeated low-level red light. J Clin Med.

[24] Gawn TJ, Samal AV, She Z (2023). The effects of intensity, spectral purity and duty cycle on red light-induced hyperopia in tree shrews. Ophthalmic Physiol Opt.

[25] Liu H, Yang Y, Guo J, Peng J, Zhao P (2023). Reitna damage after repeated low-lever red-light laser exposure. JAMA Ophthalmol.

