# Supplementary material for: A retrospective study of cumulative absolute reduction in axial length after photobiomodulation therapy
Source: BMC Ophthalmol. 2024 Apr 25;24:191. doi: 10.1186/s12886-024-03427-4 (PMC11044358; doi:10.1186/s12886-024-03427-4)
Supplement: Supplementary file 1 — Supplementary Material 1 [file 12886_2024_3427_MOESM1_ESM.docx]

### Table 3 CARE 1 frequency of each age with 1, 3, 6, 12 months PBM therapy

|  | 1M R∆0.00mm (CARE 1) | 1M R∆<-0.05mm (CARE 2) | 1M R∆<-0.10mm (CARE 3) | 1M R∆<-0.20mm(CARE 4) |
| --- | --- | --- | --- | --- |
| 4Y | 57.10% | 42.80% | 28.60% | 0.00% |
| 5Y | 60.00% | 40.00% | 30.00% | 10.00% |
| 6Y | 50.00% | 50.00% | 25.00% | 18.75% |
| 7Y | 57.50% | 42.50% | 20.00% | 7.50% |
| 8Y | 44.00% | 56.00% | 12.00% | 8.00% |
| 9Y | 38.10% | 61.90% | 23.80% | 4.80% |
| 10Y | 44.40% | 55.60% | 33.30% | 0.00% |
| 11Y | 66.70% | 33.20% | 16.70% | 0.00% |
| 12Y | 55.60% | 44.40% | 44.40% | 11.10% |
| 13Y | 0.00% | 100.00% | 100.00% | 0.00% |
|  |  |  |  |  |
|  |  |  |  |  |
|  |  |  |  |  |
|  | 3M R∆0.00mm (CARE 1) | 3M R∆<-0.05mm (CARE 2) | 3M R∆<-0.10mm (CARE 3) | 3M R∆<-0.20mm(CARE 4) |
| 4Y | 85.70% | 14.30% | 14.30% | 0.00% |
| 5Y | 56.00% | 33.00% | 44.00% | 0.00% |
| 6Y | 44.40% | 55.60% | 27.80% | 11.10% |
| 7Y | 57.50% | 42.50% | 25.00% | 7.50% |
| 8Y | 46.20% | 53.80% | 34.60% | 19.20% |
| 9Y | 38.10% | 47.60% | 38.10% | 4.80% |
| 10Y | 33.30% | 66.70% | 44.40% | 11.10% |
| 11Y | 50.00% | 50.00% | 50.00% | 12.50% |
| 12Y | 16.70% | 83.30% | 66.70% | 50.00% |
| 13Y | 100.00% | 100.00% | 100.00% | 50.00% |
|  |  |  |  |  |
|  |  |  |  |  |
|  |  |  |  |  |
|  | 6M R∆0.00mm (CARE 1) | 6M R∆<-0.05mm (CARE 2) | 6M R∆<-0.10mm (CARE 3) | 6M R∆<-0.20mm(CARE 4) |
| 4Y | 100.00% | 0.00% | 0.00% | 0.00% |
| 5Y | 50.00% | 50.00% | 50.00% | 50.00% |
| 6Y | 55.60% | 44.40% | 22.20% | 11.10% |
| 7Y | 50.00% | 50.00% | 35.00% | 15.00% |
| 8Y | 45.50% | 54.50% | 36.40% | 9.10% |
| 9Y | 22.20% | 77.80% | 55.60% | 0.00% |
| 10Y | 50.00% | 50.00% | 50.00% | 50.00% |
| 11Y | 25.00% | 75.00% | 50.00% | 25.00% |
| 12Y | 42.90% | 57.10% | 57.10% | 57.10% |
| 13Y | 0.00% | 100.00% | 100.00% | 100.00% |
|  |  |  |  |  |
|  | 12M R∆0.00mm(CARE 1) | 12M R∆<-0.05mm (CARE 2) | 12M R∆<-0.10mm (CARE 3) | 12M R∆<-0.20mm(CARE 4) |
| 4Y | 50.00% | 50.00% | 50.00% | 0.00% |
| 5Y | 0.00% | 100.00% | 0.00% | 0.00% |
| 6Y | 80.00% | 20.00% | 20.00% | 0.00% |
| 7Y | 50.00% | 50.00% | 0.00% | 0.00% |
| 8Y | 20.00% | 80.00% | 40.00% | 100.00% |
| 9Y | 25.00% | 75.00% | 25.00% | 100.00% |
| 10Y | 0.00% | 100.00% | 100.00% | 50.00% |
| 11Y | 0.00% | 100.00% | 0.00% | 0.00% |
| 12Y | 0.00% | 0.00% | 100.00% | 100.00% |
| 13Y | 0.00% | 100.00% | 100.00% | 50.00% |

Y: years old; R: right eye; CARE: cumulative absolute reduction in axial elongation
